# Suberoylanilide hydroxamic acid (SAHA) reverses chemoresistance in head and neck cancer cells by targeting cancer stem cells via the downregulation of nanog

**DOI:** 10.18632/genesandcancer.54

**Published:** 2015-03

**Authors:** Bhavna Kumar, Arti Yadav, James C. Lang, Theodoros N. Teknos, Pawan Kumar

**Affiliations:** ^1^ Department of Otolaryngology-Head and Neck Surgery; The Ohio State University Wexner Medical Center, Columbus, OH, USA; ^2^ The Ohio State University Comprehensive Cancer Center, Columbus, OH, USA

**Keywords:** HDAC, SAHA, Cancer Stem Cells, Chemoresistance, Tumorsphere, Nanog, Human papillomavirus (HPV)

## Abstract

Acquisition of chemoresistance and metastatic phenotype are the major causes of treatment failure and mortality in head and neck squamous cell carcinoma (HNSCC) patients. Histone deacetylases (HDACs) have been shown to be overexpressed in many tumor types and directly linked to poor prognosis. In this study, we demonstrate that HDACs are markedly elevated in HNSCC. HDACs expression was further increase in cisplatin resistant cell lines (CisR). In addition, cisplatin-resistant cells showed enhanced stem cell properties and tumor metastasis. Depletion of HDAC1 and 2 in CisR cell lines significantly reversed cisplatin resistance and tumorsphere formation. Next, we tested the efficacy of Suberoylanilide hydroxamic acid (SAHA), an HDAC inhibitor, by using both *in vitro* and *in vivo* models. SAHA significantly inhibited cell proliferation and synergistically enhanced the anti-proliferative effects of cisplatin. In addition, SAHA significantly decreased tumorsphere formation by markedly reducing nanog expression. In a SCID mouse xenograft model, SAHA significantly enhanced the anti-tumor effects of cisplatin treatment with no added systemic toxicity. Furthermore, SAHA and cisplatin combination treatment significantly decreased tumor metastasis and nanog expression, *in vivo*. Taken together, our results suggest that targeting HDACs with SAHA could be an effective treatment strategy for the treatment of HNSCC patients.

## INTRODUCTION

Head and neck squamous cell carcinoma (HNSCC) is the 8th leading cancer worldwide with almost 650,000 new cases diagnosed every year and 350,000 cancer-related deaths annually [1-3]. Although advancements in the anti-cancer treatments including surgery, radiation and chemotherapy have increased the local control of HNSCC, the overall survival rates have not improved significantly over the last three decades [4, 5]. Five year survival rates for patients with early stage localized head and neck cancers are more that 80% but drop to 40% when the disease has spread to the neck nodes, and to below 20% for patients with distant metastatic disease [4]. Cisplatin is one of the most widely used chemotherapeutic agent for the treatment of head and neck cancers [6]. However, despite an initial favorable response, many patients acquire resistance to it leading to treatment failures [7]. Therefore, it is important to understand the molecular mechanisms that contribute to drug resistance in order to identify novel therapeutic targets for head and neck cancer.

Recent studies have highlighted the role of histone deacetylases (HDACs) in regulating a number of genes that are involved in both cancer initiation and cancer progression [8-16]. Chromatin configuration is tightly regulated by histone acetylation and deacetylation process and this balance between histone acetylation and deacetylation determines the level at which a particular gene is transcribed [17]. Histone acetylation by histone acetyltransferase (HAT) relaxes the chromatin, thereby allowing transcription factors and RNA polymerase II recruitment. HDACs remove the acetyl groups from histones, creating a non-permissive chromatin that represses gene transcription [17]. Importantly, most of the genes repressed by HDACs are tumor suppressors, cell cycle regulators or apoptosis inducers [18]. HDACs also bind and deacetylate a number of other proteins including transcription factors [18]. Recent studies have shown that HDACs can regulate the self-renewal of cancer stem cells by modulating nanog expression [19, 20]. In humans, 18 HDACs have been identified and grouped in four classes; class I comprises of HDAC1-3 and 8; class II comprises HDACs 4-7, 9 and 10; class III comprises the NAD+-dependent HDACs (SIRT1-7) and class IV comprises HDAC11, which has some features of both class I and Class II HDACs [21, 22].

Several inhibitors of HDACs have been developed in the last decade and many HDAC inhibitors are in clinical trials for the treatment of solid and hematological malignancies [23-25]. Vorinostat (Suberoyanilide hydroxamic acid, SAHA) is one of the most advanced pan-HDAC inhibitor that was approved by FDA in 2006 for the treatment of cutaneous T-cell lymphoma [26]. SAHA was initially developed as a derivative of hexamethylene bisacetamide (HMBA) to treat myelodysplatic syndrome and acute myelodysplastic leukemia [27]. Recent studies have shown that SAHA has significant anti-cancer activities against many solid tumors [23, 26]. SAHA has been shown to inhibit all 11 known human class I and II HDACs [28]. In addition to inhibiting the activity of HDACs, SAHA is also known to regulate the expression of a number of other proteins including transcription factors, pro-apoptotic proteins and anti-apoptotic proteins [29].

In this report, we demonstrate that the expression of HDAC1, 2 and 6 is significantly upregulated in head and neck cancer cells. In addition, HDACs expression is further increased in cisplatin resistant (CisR) cells. HDAC1 and 2 knockdown in cisplatin-resistant cell lines by siRNA significantly reversed cisplatin resistance and tumorsphere formation. Similarly, treatment with pan-HDAC inhibitor SAHA significantly enhanced the anti-tumor effects of cisplatin in a synergistic manner. In addition, SAHA treatment significantly decreased tumorsphere formation and markedly reduced nanog and survivin expression. Similar to the *in vitro* results, SAHA and cisplatin combination treatment significantly decreased tumor growth and tumor metastasis, *in vivo*.

## RESULTS

### HDAC1 expression is significantly higher in primary tumor samples and cancer cell lines from head and neck cancer patients

**Figure 1:**
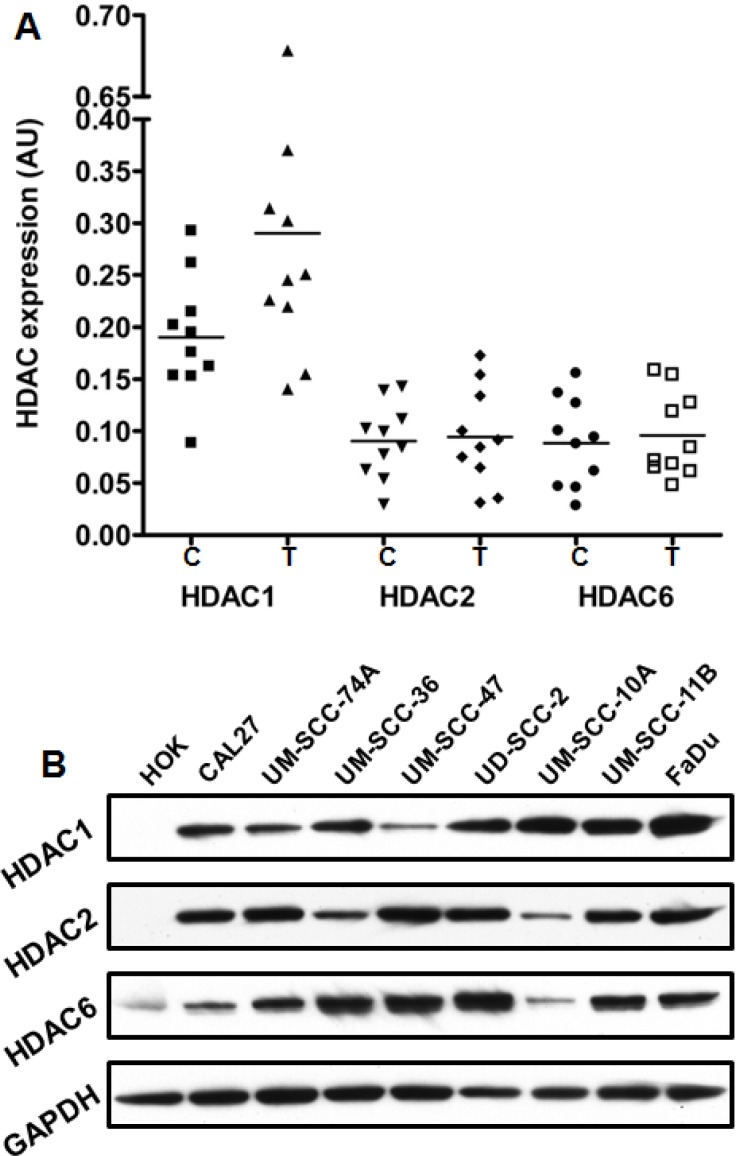
The expression of HDACs is upregulated in HNSCC patient's primary tumors and cell lines A: HDAC1, HDAC2 and HDAC6 expression in 10 primary tumor samples (T) and in 10 adjacent normal tissue (C) of head and neck cancer patients was analyzed by quantitative real-time PCR (RT-PCR) and statistical significance was analyzed by Mann-Whitney test. B: HDAC1, HDAC2 and HDAC6 expression was analyzed in 8 head and neck cancer cell lines by Western blotting and compared to the expression in normal human oral keratinocytes (HOK). Equal protein loading was verified by stripping the blots and reprobing with GAPDH antibody.

We measured HDAC1, HDAC2 and HDAC6 levels in 20 frozen tissue samples from head and neck cancer patients (10 tumor and 10 adjacent normal controls) by real-time PCR. Our results show that HDAC1 expression is significantly higher (Mann-Whitney test; p = 0.0262) in head and neck tumors as compared to adjacent normal control tissues (Fig. 1A). However, we did not observe any significant difference in HDAC2 and HDAC6 levels in tumor samples and adjacent normal controls. We next examined HDAC1, HDAC2 and HDAC6 levels in 8 head and neck cancer cell lines and normal human oral keratinocytes (HOK). All the three HDACs were markedly upregulated in head and neck cell lines as compared to HOK (Fig. 1B).

### HDACs expression is markedly upregulated in cisplatin-resistant head and neck cancer cells

**Figure 2:**
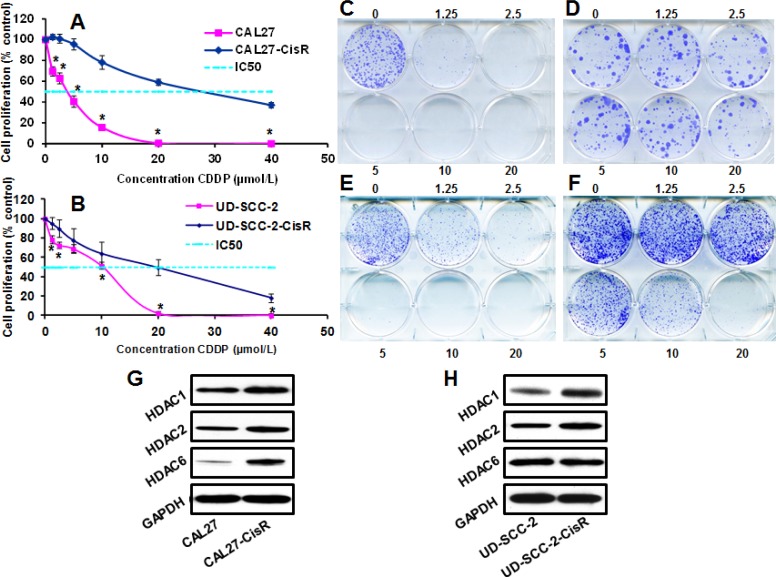
The expression of HDACs is upregulated in cisplatin-resistant cells A-B: CAL27-CisR and its parental cell line CAL27 (A) or UD-SCC-2-CisR and its parental cell line UD-SCC-2 (B) were treated with different concentrations of cisplatin (CDDP) and cell proliferation was assessed by MTT assay. C-F: CAL27 (C) and CAL27-CisR (D) or UD-SCC2 (E) and UD-SCC-2-CisR (F) were treated with different concentrations of CDDP (0-20 μmol/L) and colony formation was examined by culturing tumor cells in 6-well plates for 10 days. G-H: HDAC1, HDAC2 and HDAC6 expression was analyzed by Western blotting in CAL27 and CAL27-CisR cells (G) or UD-SCC-2 and UD-SCC-2-CisR cells. Equal protein loading was verified by stripping the blots and reprobing with GAPDH antibody.

To examine the role of HDACs in the acquisition of cisplatin resistance, we took two head and neck cancer cell lines (one HPV-negative, CAL27 and one HPV-positive, UD-SCC-2) that are relatively sensitive to cisplatin treatment (CAL27, IC_50_ 3 μmol/L and UD-SCC-2, IC_50_ 10 μmol/L) and induced cisplatin resistance by culturing these cell lines in increasing doses of cisplatin over an extended period of time. These new cisplatin resistant cell lines, designated CAL27-CisR (IC_50_ 28 μmol/L) and UD-SCC-2-CisR (IC_50_ 20 μmol/L), were significantly more resistant to cisplatin as compared to their parental cell lines (Fig. 2A-F). Both of these cisplatin resistant cells also showed marked increase in HDAC1 and HDAC2 expression, whereas HDAC6 expression was increased in CAL27-CisR cells only (Fig. 2G-H).

### Cisplatin-resistant cells show increased stemness properties and tumor metastasis

**Figure 3:**
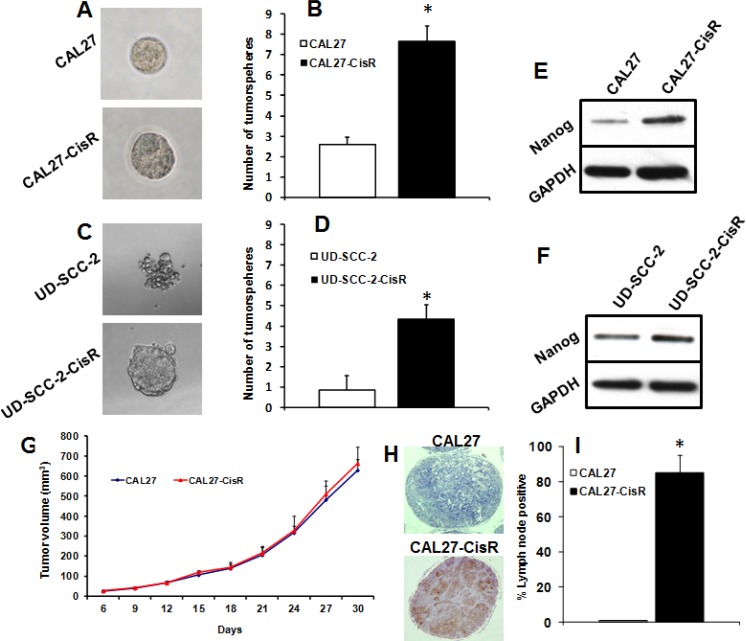
Cisplatin-resistant cells show increased stemness properties and metastasis A-D: CAL27 and CAL27-CisR cells (A-B) or UD-SCC-2 and UD-SCC-2-CisR cells (C-D) were cultured in ultra-low adhesion plates to examine tumorsphere formation efficiency. *, represent a significant higher tumorsphere numbers as compared to control. E-F: Nanog expression was analyzed in CAL27 and CAL27-CisR (E) or UD-SCC-2 and UD-SCC-2-CisR (F) by Western blotting. G-I: Tumor cells (CAL27 and CAL27-CisR) were implanted in the flanks of SCID mice and tumor growth and tumor metastasis to draining lymph nodes was analyzed. G: Tumor growth curves for CAL27 and CAL27-CisR tumors. H-I: Lymph nodes were stained with pan-cytokeratin antibody and analyzed for metastatic disease. *, represent significant higher lymph nodes with metastatic disease as compared to control.

We next examined if induction of cisplatin-resistance in head and neck cancer cells could enhance stemness properties. The capacity of tumor cells to form tumorsphere is often used as a surrogate measure of stemness in cancer cells. CAL27-CisR and UD-SCC-2-CisR cells and their parental cell lines (CAL27 and UD-SCC-2) were cultured in ultralow binding plates for 10 days and tumorsphere formation was quantified. Cisplatin-resistant cells (CAL27-CisR and UD-SCC-2-CisR) showed significantly higher tumorsphere formation as compared to their parental cell lines (CAL27 and UD-SCC-2, Fig. 3A-D). Nanog is one of the key transcription factors that has been shown to promote cancer progression by regulating cancer stem cells [30, 31]. We next examined if induction of cisplatin resistance upregulated nanog expression in head and neck cancer cells. Indeed, nanog expression was markedly upregulated in both CAL27-CisR and UD-SCC2-CisR cells (Fig. 3E-F). We next used a SCID mouse model to examine if cisplatin-resistant cells show higher tumor metastasis. We did not observe any significant difference in tumor growth pattern in CAL27-CisR and its parental cell line (Fig. 3G). Interestingly, lymph nodes from animal carrying CAL27 tumors were negative for metastatic disease whereas 80% of lymph nodes from animal carrying CAL27-CisR tumor were positive for metastatic disease (Fig. 3H-I).

### HDAC1/2 knockdown significantly reverses cisplatin resistance and tumorsphere formation

**Figure 4:**
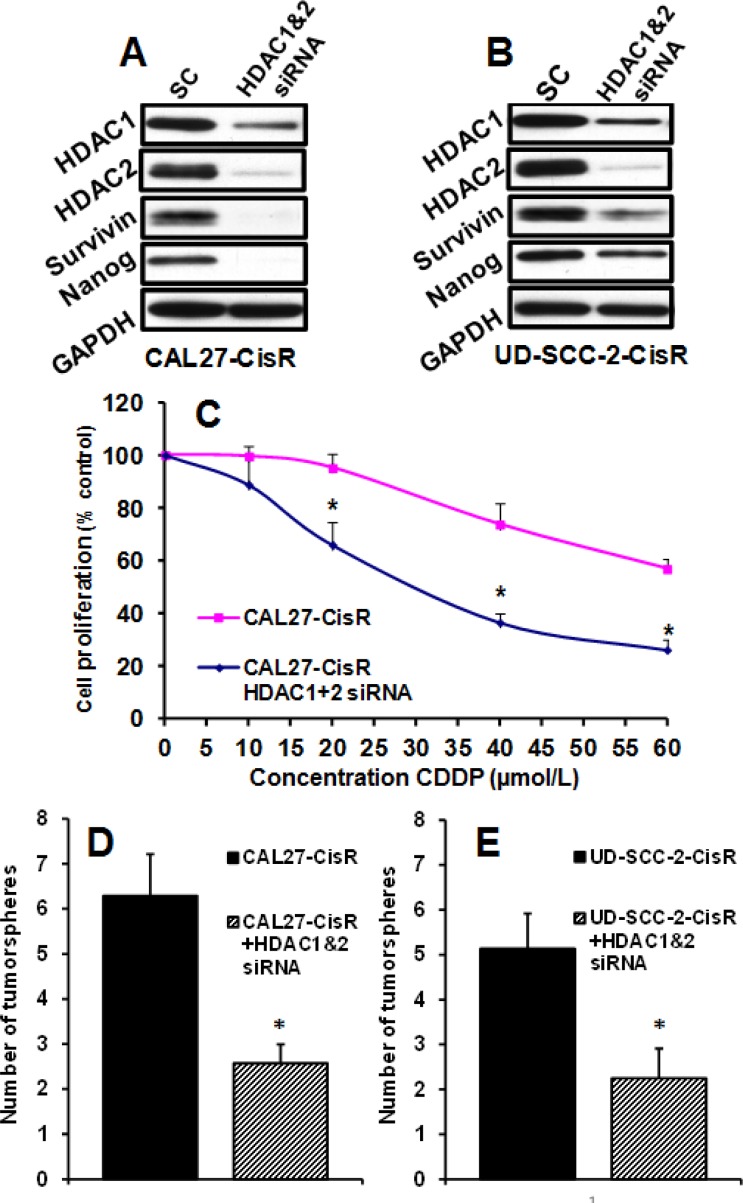
HDAC1/2 knockdown by siRNA significantly reverses cisplatin resistance and tumorsphere formation HDAC1 and HDAC2 were knocked down in CAL27-CisR and UD-SCC-2-CisR cells by siRNA. A-B: HDAC1 and HDAC2 knockdown was verified by Western blotting. Survivin and nanog expression was examined in HDAC1&2 knockdown cells. C: HDAC1&2 were knocked down in CAL27-CisR cells and cell proliferation examined by MTT. D-E: Tumorsphere formation efficiency was examined in CAL27-CisR and UD-SCC-2-CisR cells after knocking down HDAC1&2. *, represent a significant higher tumorsphere numbers as compared to control.

To further understand the role of HDACs in chemoresistance and stem cell function, we knocked down HDAC1 and HDAC2 in cisplatin resistant cell lines (Fig. 4A-B). HDAC1&2 knockdown significantly reversed resistance in cisplatin-resistant CAL27-CisR cells (Fig. 4C). Similarly, HDAC1&2 knockdown significantly decreased tumorsphere formation in cisplatin-resistant cell lines (Fig. 4D-E). We have recently shown that survivin is one of the key molecules that regulate cisplatin resistance [32]. We next examined if HDAC1&2 knockdown reverses cisplatin resistance by downregulating survivin expression. Indeed, HDAC1&2 knockdown markedly decreased survivin levels in CAL27-CisR and UD-SCC-2-CisR cells (Fig. 4 A-B). In addition, HDAC1&2 knockdown also markedly downregulated nanog expression (Fig. 4 A-B).

### HDAC inhibitor SAHA significantly reverses cisplatin resistance in head and neck cancer cells

**Figure 5:**
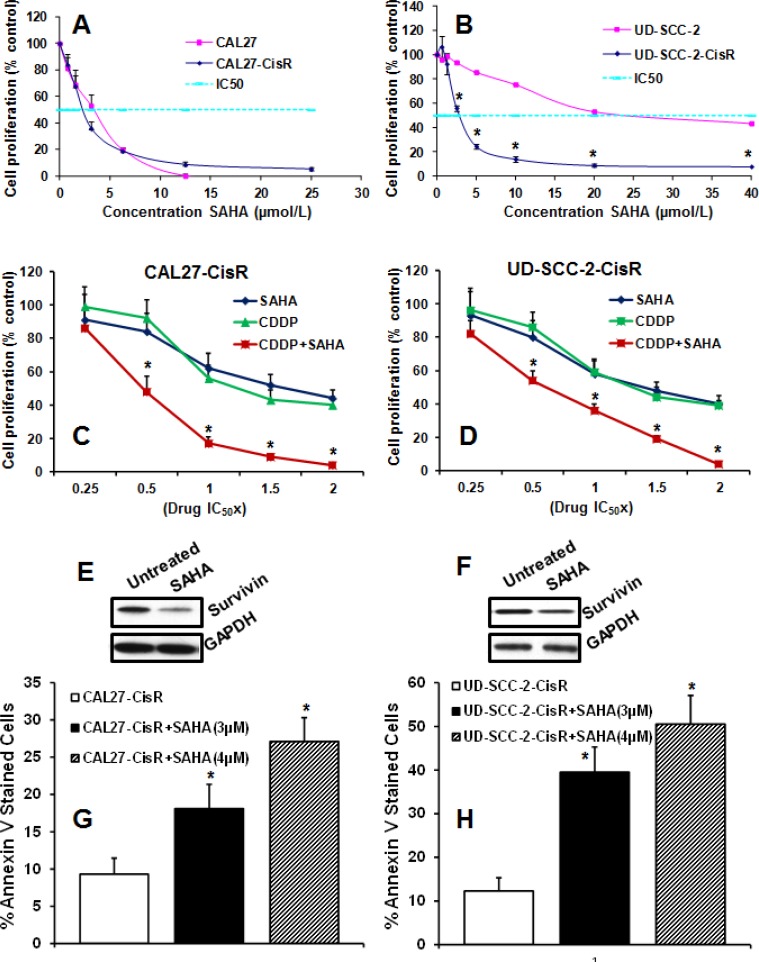
SAHA significantly reverses cisplatin resistance A-B: CAL27-CisR and its parental cell line CAL27 (A) or UD-SCC-2-CisR and its parental cell line UD-SCC-2 (B) were treated with different concentrations of SAHA and cell proliferation was assessed byMTT assay. C-D: CAL27-CisR (C) or UD-SCC-2-CisR (D) cells were treated with SAHA or cisplatin (CDDP) alone or in combination at 0.25, 0.5, 1, 1.5 and 2 times their respective IC_50_ doses and cell proliferation was assessed by MTT assay. Results were analyzed according to Chou-Talalay method and the combination index (CI) values calculated by CompuSyn software. E-F: CAL27-CisR (E) or UD-SCC-2-CisR (F) cells were treated with SAHA and survivin expression was examined by Western blotting. G-H: CAL27-CisR (G) and UD-SCC-2-CisR (H) cells were treated with SAHA (3 μM and 4 μM) and tumor cell apoptosis was analyzed by annexin V staining.

We next examined if SAHA could reverse cisplatin resistance in head and neck cancer cells and enhance its anti-tumor efficacy in a synergistic manner. SAHA treatment was highly effective in inhibiting tumor cell proliferation in both the cisplatin-resistant head and neck cancer cell lines (CAL27-CisR and UD-SCC-2-CisR) in a dose dependent manner (Fig. 5A-B). Interestingly, SAHA treatment was significantly more effective in cisplatin-resistant HPV-positive UD-SCC-2-CisR cells as compared to its parental counterpart (Fig. 5B). In the next set of experiments, we examined if SAHA could enhance the anti-proliferative effect of cisplatin (CDDP) in a synergistic manner. The SAHA and cisplatin combination synergy was measured by calculating the combination index (CI) according to Chou-Talalay method [33] using a fixed dose ratio. Both SAHA and CDDP were added to the tumor cell cultures at 0.25x, 0.5x, 1x, 1.5x and 2x their respective IC _50_ doses. Cell proliferation in both the cell lines were markedly decreased following combination treatment at the multiple paired concentrations as compared to treatment with either of single agents alone (Fig. 5C-D). Combination index (CI) for different effective doses (ED) was calculated using CompuSyn software. CI values for CAL27-CisR cells at ED50, ED75 and ED90 were 0.587, 0.434 and 0.410 respectively. The CI values for UD-SCC-2-CisR cells were 0.770, 0.510 and 0.411 at ED50, ED75 and ED90, respectively. These results suggest that SAHA and cisplatin combination treatment was highly effective in inhibiting tumor cell proliferation in a synergistic manner in both the cisplatin resistant cell lines.

Recently, we have shown that survivin is markedly upregulated in cisplatin-resistant cells and mediates chemoresistance [32]. Interestingly, SAHA treatment markedly decreased survivin levels in both cisplatin-resistant cell lines (Fig. 5E-F). Survivin is an important cell survival protein and we next examined if SAHA-mediated downregulation of survivin enhances tumor cell apoptosis. Indeed, SAHA treatment significantly enhanced tumor cell apoptosis in a dose dependent manner (Fig. 5G-H).

### SAHA significantly decreases tumorsphere formation and nanog expression

**Figure 6:**
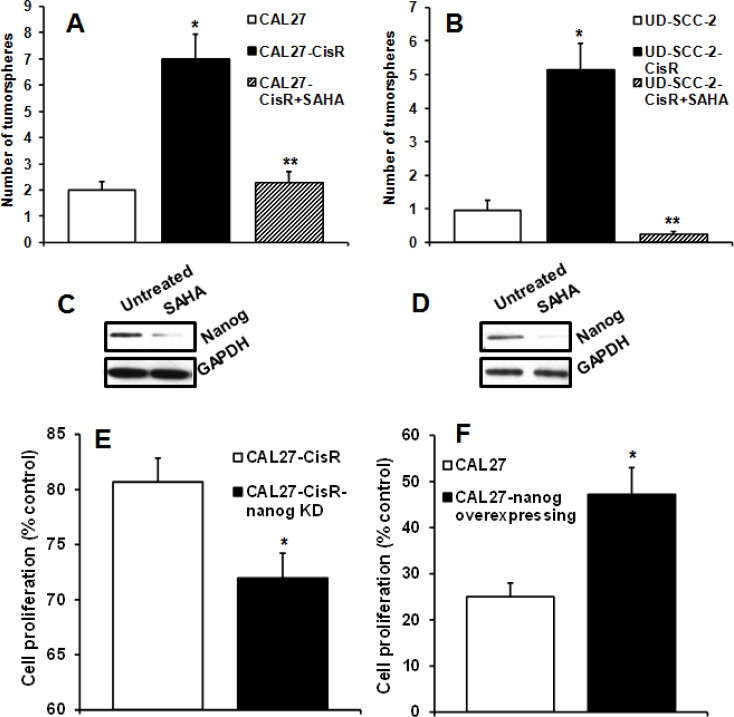
SAHA significantly reverses cisplatin resistance and reduces tumorsphere formation A-B: CAL27-CisR (A) or UD-SCC-2-CisR (B) cells were treated with SAHA and tumorsphere formation efficiency calculated. *, represent a significant increase in tumorsphere number and **, represent a significant decrease in tumorsphere. C-D: Nanog expression was examined in CAL27-CisR (C) and UD-SCC-2-CisR (D) cells by Western Blotting. E: Nanog was knocked down in CAL27-CisR cells and cell proliferation examined in response to cisplatin treatment (10 μM). F: Nanog was overexpressed in CAL27 cells and cell proliferation examined in response to cisplatin treatment (10 μM).

We next investigated the effects of SAHA treatment on tumorsphere formation. SAHA treatment significantly decreased tumorsphere formation in both the HPV-positive and HPV-negative cisplatin-resistant cell lines (Fig. 6A-B). Since both CAL27-CisR and UD-SCC-2-CisR cells showed a marked increase in nanog expression (Fig. 3E-F), we examined if SAHA decreased tumorsphere formation by downregulating nanog expression. SAHA treatment markedly decreased nanog levels in CAL27-CisR and UD-SCC-2-CisR cells (Fig. 6 C-D). We next examined if enhanced nanog expression in cisplatin resistant cells may be contributing to chemoresistance. To answer this question, we used two strategies. In the first set of experiments, we knocked down nanog in cisplatin-resistant cells by siRNA and examined cell proliferation in response with cisplatin treatment. Nanog knockdown significantly reversed chemoresistance in CAL27-CisR cells (Fig. 6E). In the second experiment, we overexpressed nanog in CAL27 (cisplatin-sensitive cell line) and examined cell proliferation in response to cisplatin treatment. Nanog overexpression significantly increased chemoresistance in CAL27 cells (Fig. 6F)

### SAHA and cisplatin combination treatment significantly decreases tumor growth and tumor metastasis

**Figure 7:**
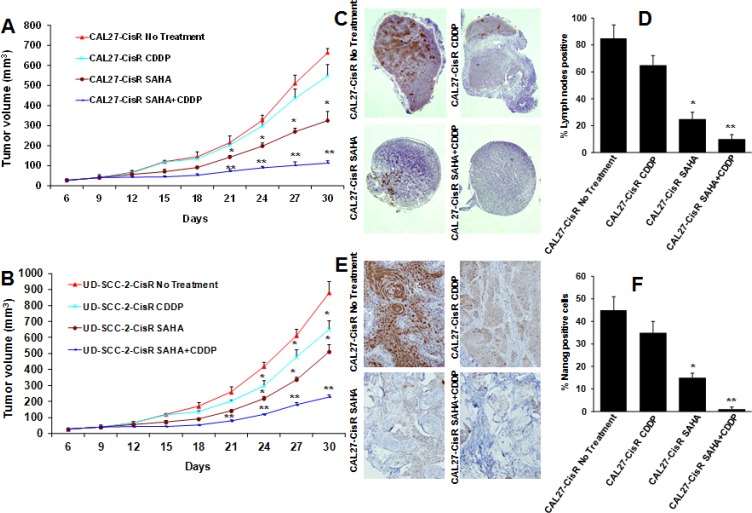
SAHA and cisplatin treatment significantly decrease tumor growth and tumor metastasis Animals bearing CAL27-CisR and UD-SCC-2-CisR tumors were treated with SAHA (50 mg/kg/twice a week) or cisplatin (CDDP, 5 mg/kg, twice a week) alone or in combination. A-B: Tumor growth curves for CAL27-CisR (A) and UD-SCC-2-CisR (B). *, represents a significant difference (p<0.05) as compared to no treatment group and **, represents a significant difference (p<0.05) as compared to single treatment groups. C-D: Lymph nodes were stained for metastatic disease with pan-cytokeratin antibody. C: Representative photomicrographs of lymph nodes from animals bearing CAL27-CisR tumors and treated with SAHA or cisplatin alone or in combination. D: Percentage of lymph nodes positive for tumor cell metastasis. E-F: CAL27-CisR tumor samples were stained for nanog expression and percentage of nanog positive cells quantified. *, represents a significant difference (p<0.05) as compared to no treatment group and **, represents a significant difference (p<0.05) as compared to SAHA alone.

Our *in vitro* data suggest that SAHA is a potent inhibitor of head and neck cancer cell proliferation and it enhances the anti-tumor effects of cisplatin therapy in a synergistic manner. We further validated our *in vitro* results by using a SCID mouse xenograft model. In the first set of experiments, we used a HPV-negative (CAL27-CisR) cell line. Cisplatin (CDDP, 5 mg/kg/twice a week) and SAHA (50 mg/kg/twice a week) treatment alone showed 21% and 48% tumor growth inhibition at day 30, respectively (Fig 7A). SAHA in combination with cisplatin showed significantly higher tumor growth inhibition as compared to untreated group (85%) or single agent alone (Fig 7A). In addition, the combination treatment was very well tolerated, and it did not cause any animal toxicity or induce significant decrease in body weight. In the second set of experiments, we used HPV-positive (UD-SCC-2-CisR) cell line. Cisplatin (CDDP, 5 mg/kg/twice a week) and SAHA (50 mg/kg/twice a week) treatment alone showed 27% and 45% tumor growth inhibition at day 30, respectively (Fig 7B). As observed with CAL27-CisR cells, SAHA and cisplatin combination treatment was most effective in inhibiting tumor growth of UD-SCC-2-CisR tumors (79%, Fig. 7B).

We next examined the effect of SAHA and cisplatin combination treatment on tumor metastasis. At the end of tumor growth study (day 30), lymph nodes from SCID mice were removed and analyzed for metastatic disease by staining with pan-cytokeratin antibody. Eighty five percent of the draining lymph nodes from untreated CAL27-CisR bearing animals were positive for metastatic disease (Fig. 7C-D). Animals treated with cisplatin and SAHA alone showed significant decrease in draining lymph nodes positive for metastatic disease (65% and 25% respectively, Fig. 7C-D). SAHA and cisplatin combination treatment was most effective in inhibiting tumor metastasis (10% nodes positive).

Our *in vitro* results showed that nanog, a key transcription factor that regulates pluripotency of cancer stem cells, is markedly upregulated in cisplatin-resistant head and neck cancer cells. We next examined if SAHA and cisplatin combination treatment downregulated nanog expression, *in vivo*. Indeed, SAHA and cisplatin combination treatment significantly downregulated nanog expression *in vivo* (Fig. 7 E-F).

## DISCUSSION

Despite advancements in the techniques for surgery, radiation and chemotherapy, the overall survival rates for patients with HNSCC have not significantly improved over the three decades [1, 34]. This could be due to the acquisition of chemo and radio-resistance that in turn leads to local and distant failure [35]. Therefore, there is an urgent need to identify new therapeutic targets so that novel treatment regimens can be developed to improve the therapeutic efficacy. HDACs could be a potential target for the treatment of head and neck cancer patients as overexpression of HDACs has been observed in tumors from head and neck cancer patients and HDACs expression is linked to poor prognosis [36, 37]. In our study, we also observed significantly higher levels of HDAC1 in primary tumors from head and neck cancer patients as compared to surrounding normal tissue. In addition, HDAC1, 2 and 6 levels were markedly upregulated in head and neck cancer cell lines. More importantly, HDACs levels were further elevated in cisplatin resistant cells as compared to their parental cisplatin sensitive cells. Therefore, we hypothesized that targeting HDACs in advanced HNSCC could reverse the resistant phenotype in tumor cells, thereby enhancing the therapeutic efficacy of cisplatin. To test this hypothesis, we carried out combination treatment studies *in vitro* and *in vivo*, by using our SCID mouse models. We selected SAHA, a pan-HDAC inhibitor for this study as it has been shown to demonstrate a potent antitumor activity against both hematologic and solid tumors [26, 38]. In addition, SAHA has been successfully used in clinics for the treatment of cutaneous T-cell lymphoma and it has been found to be very well tolerated [39].

Patients with head and neck cancer encompass a heterogeneous group and can be further subdivided into two distinct tumor subtypes; human papillomavirus (HPV) negative and HPV-positive tumors. Majority of HNSCC patients with HPV-positive tumors respond very well to traditional chemo-radiotherapy and demonstrate significantly favorable clinical outcomes [40, 41]. However, there is a small subset of HPV-positive patients that do not respond well to standard therapy and show markedly poor clinical outcome [42, 43]. In contrast to HPV-positive patients, majority of HPV-negative patients are usually smokers, have more aggressive disease, and many of these patients develop resistance to chemotherapy leading to poor prognosis. In order to mimic cisplatin-resistant phenotype, we generated a cisplatin resistant HPV-negative cell line in our laboratory by culturing a cisplatin sensitive tongue SCC cell line CAL27 in increasing doses of cisplatin over a period of time [32]. Similarly, we generated cisplatin resistant HPV-positive cell line (UD-SCC-2-CisR) in our laboratory. To our knowledge, this is the first cisplatin resistant HPV-positive cell line generated in the laboratory and could be very important to test the efficacy of novel therapies in pre-clinical studies.

In our study, SAHA treatment was very effective in inhibiting tumor cell proliferation and tumorsphere formation in both the cisplatin resistant HPV-negative (CAL27-CisR) as well as HPV-positive (UD-SCC-2-CisR) cell lines. UD-SCC-2-CisR contains wild-type p53, whereas CAL27-CisR has mutant p53 gene. Interestingly, both these cell lines were equally sensitive to SAHA treatment regardless of p53 mutational status, thereby suggesting that SAHA mediates its anti-tumor effects independent of p53 status. This is important as more that 50% of head and neck tumors have mutant p53 and some of the targeted inhibitors selectively inhibit cell growth in cancer cells with wild-type p53 only [44, 45]. Combination index analysis by Chou-Talalay method [33] demonstrated that SAHA and cisplatin combination treatment is highly effective and synergistic in mediating anti-tumor effects. These potent anti-tumor effects of SAHA could be due to its inhibitory effects on a number of key anti-apoptotic members of Bcl-2 and IAP family particularly survivin [23, 24, 26]. We have previously shown that survivin levels are markedly upregulated in cisplatin-resistant cells and survivin knockdown with siRNA or treatment with survivin inhibitor YM155 significantly reverses cisplatin resistance in the cisplatin-resistant cells [32]. In addition to reversal of chemoresistance, we also observed a significant decrease in tumorsphere formation and nanog expression in SAHA treated cells. Nanog is a key stem cell transcription factor that has been shown to be regulated through HDACs [19, 20, 30]. Nanog is shown to be enriched in cancer stem cell population and has also been shown to mediate cisplatin resistance in cancer cells [46, 47]. Our study demonstrates for the first time that SAHA treatment can decrease nanog expression in cisplatin resistant cells and reverse chemoresistance in these cells. In addition, SAHA-mediated downregulation of nanog expression might also be blocking tumor metastasis by decreasing cancer stem cell population [48]. Recently, a HDAC inhibitor romidepsin was tested in a phase II clinical trial in patients with recurrent/metastatic head and neck cancer and showed limited activity as a single agent [49]. The results from our study provide a scientific rationale to test SAHA in a combination regimen to reverse chemoresistance and enhance the therapeutic efficacy of cisplatin treatment. At our institution, we are currently conducting a phase I clinical trial with SAHA in combination with standard chemo-radiation regimen for the treatment of advanced head and neck cancer patients.

In conclusion, we have demonstrated that SAHA significantly enhances the anti-tumor effects of cisplatin treatment in both HPV-negative and HPV-positive cancers. This is important because it is projected that HPV-positive cancers will constitute the majority of head and neck cancer in the United States in the near future [50]. Therefore, targeting HDACs could be a useful strategy for this patient population as well as for patients with HPV-negative HNSCC that do not respond to current chemotherapy.

## MATERIALS AND METHODS

### Patient samples, cell culture and Reagents

Use of patient samples was approved by The Ohio State University institutional review board. Tumor and adjacent normal tissue samples were collected from head and neck cancer patients undergoing surgical resection at the James Cancer Center and Solove Research Institute. Normal samples were collected from areas adjacent to the tumor but outside the tumor margins (patient tumor characteristics are presented in Supplementary Table S1). The human HNSCC cell lines CAL27 and FaDu were purchased from the ATCC (Manassas, VA, USA). UM-SCC-74A, UM-SCC-36, UM-SCC-47, UM-SCC-10A and UM-SCC-11B were obtained from Dr. Thomas E. Carey (University of Michigan). UD-SCC-2 cell line was obtained from Dr. Henning Bier at University of Munich, Germany. All tumor cell lines were cultured in DMEM supplemented with 10% fetal bovine serum, non-essential amino acids and penicillin/streptomycin. The identity of all cell lines was confirmed by STR genotyping (Identifier Kit, Applied Biosystems, Carlsband, CA).

The characteristic of cell lines [51, 52] (origin, p53 status, HPV status) are presented in Supplementary Table S2. Normal human oral keratinocytes (HOK) were purchased from ScienCell (Carlsbad, CA). Cisplatin (CDDP) was purchased from Sigma-Aldrich (St. Louis, MO) and suberoylanilide hydroxamic acid (SAHA) was purchased from Selleck Chemicals (Houston, TX). Antibodies against survivin and HDAC6 were obtained from Cell Signaling Technology (Danvers, MA), antibodies against HDAC1 and HDAC2 from Abcam (Cambridge, MA) and pan-cytokeratin antibody from Santa Cruz Biotechnology (Santa Cruz, CA). Nanog antibody for Western blotting from Novus Biologicals (Littleton, CO) and for immunohistochemistry was obtained from Abcam (Cambridge, CA). GAPDH antibody was obtained from EMD Millipore (Billerica, MA).

### Quantitative Real-Time PCR

RNA from the HNSCC tumors and adjacent normal controls was extracted using TRIzol reagent (Invitrogen). RNA was transcribed into cDNA and PCR amplified for gene transcripts using TaqMan primer/probes (HDAC1 Hs02621185_s1, HDAC2 Hs00231032_m1, HDAC6 Hs00195869_m1; Applied Biosystems). Relative changes in transcript levels were determined using the 2 (−ßßCt) method [53] using *OAZ1* or *β-actin* as reference housekeeping genes.

### Induction of cisplatin resistance in head and neck cancer cell lines

Parental cell lines CAL27 and UD-SCC-2 were grown in increasing concentrations of cisplatin over 6 months to generate cisplatin-resistant variants (CAL27-CisR and UD-SCC-2-CisR) [32]. The parental cell lines as well as the cisplatin-resistant variants (CisR) were genotyped by STR profiling.

### Cell Proliferation Assay

The sensitivity of cells to cisplatin and SAHA was measured using the MTT-based colorimetric cell proliferation kit (Roche Applied Science, Mannheim, Germany) [32]. The percentage cell growth inhibition for each treatment group was calculated by adjusting the untreated control group to 100%. Data were analyzed using GraphPad Prism software (GraphPad Sofware, Inc., San Diego, CA) and the dose response curves were used to calculate the concentration of SAHA or cisplatin resulting in 50% inhibition of cell proliferation (IC50) using a four parametric logistical model. All experiments were repeated at least 3 times.

For drug combination studies, the synergistic effect was assessed by the combination index (CI), according to the method of Chou and Talalay wherein synergism is defined as CI<1, while antagonism is CI>1, and an additive effect is considered as CI=1 [33]. The CI values were calculated using CompuSyn software (ComboSyn, Inc., Paramus, NJ).

### Western Blot Analysis

Whole cell lysates were run on NuPAGE Bis-Tris gels (Invitrogen) under reducing conditions, blotted onto PVDF membranes (GE Healthcare Life Sciences/Amersham, Piscataway, NJ), probed with primary antibodies, then rinsed and incubated with sheep anti-mouse or donkey anti-rabbit conjugated with horseradish peroxidase (GE Healthcare). The membranes were visualized using the ECL western blotting substrate (Pierce, Rockford, IL) according to the manufacturer's instructions.

### HDAC1, HDAC2 and nanog knockdown by siRNA

CA27-CisR and UD-SCC-2-CisR cells were transfected with HDAC1, HDAC2 or nanog siRNA (siGENOME SMARTpool, GE Dharmacon) using Lipofectamine 2000 as per the manufacturer's protocol. Seventy two hours post transfection, cells were either used for cell proliferation, tumorsphere formation or whole cell lysates were prepared for Western blotting.

### Generation of Nanog overexpressing cells

Human nanog gene was stably overexpressed in tumor cells using lentiviral vector [54]. Nanog construct along with retroviral packaging and envelope constructs were introduced into 293T cells (Invitrogen) using Lipofectamine 2000 (Life Technology, Grand Island, NY). Viral supernatants were collected after 24 hours, centrifuged and filtered. Tumor cells were transduced with nanog or control vector by overnight incubation with viral supernatants in the presence of 4 μg/ml polybrene. Positive (stable) tumor cell clones were selected using puromycin (2 μg/ml). Nanog overexpression was confirmed by Western blotting.

### Colony formation assay

Cells were seeded in 6-well plates (CAL27, CAL27-CisR: 1000 cells; UD-SCC-2, UD-SCC-2-CisR: 3000 cells) and allowed to adhere overnight. Cells were then treated with different concentrations of cisplatin and ultured for 10 days. Colonies were fixed with ice-cold methanol and stained with 0.5% crystal violet solution.

### Tumorsphere Forming Assays

Tumor cells were cultured in 6-cm ultralow adhesion culture dish (Corning, PA, USA) containing serum free DMEM/F-12 media supplemented with N2, B-27 (Invitrogen), 10 ng/ml EGF, 10 ng/ml basic-FGF (PeproTech, Rocky Hill, NJ) and antibiotic-antimycotic (Life Technologies). After 10 days, tumorspheres (>50 μm) were counted.

### SCID mouse xenograft model

6-8 week old SCID mice (NCI) were used in all the *in vivo* experiments [55]. Tumor cells (1 × 106) were mixed with 100 μl of Matrigel and injected in the flanks of SCID mice. After 8 days, mice were stratified into different groups (n=5), so that the mean tumor volume in each group was comparable. At days 8, 11, 14, 17, 21, 24 and 28 animals were treated with SAHA (50 mg/kg) or cisplatin (5 mg/kg) via I.P. injections. Tumor volume measurements [volume (mm3) = L × W2/2 (length L, mm; width W, mm)] began on day 6 and continued twice a week until the end of the study. After 36 days, primary tumors and draining lymph nodes were carefully removed and analyzed for naong expression and metastatic disease respectively.

### Immunohistochemistry

The xenograft tumor tissues and lymph nodes were fixed in 4% paraformaldehyde overnight and paraffin embedded. Tissue sections were deparffinized and pretreated with antigen retrieval buffer [32]. Endogenous peroxidase and non-specific binding sites were blocked and the sections were incubated with anti-pan-cytokeratin or anti-nanog antibodies. Primary antibody binding was detected using the reagents from the Vetastain Elite ABC Kit (Vector Laboratories, Burlingame, CA). The immunohistochemistry images were captured using Nikon Eclipse 80i microscope with DS-Ri1 camera (Nikon, Melville, NY).

### Statistical Analysis

Data from all the experiments are expressed as mean + SEM from a minimum of 3 independent experiments. The HDACs expression in patient samples was analyzed by Mann-Whitney test. IC_50_ values for SAHA and cisplatin for tumor cell proliferation inhibition was calculated using GraphPad Prism software (GraphPad Sofware, Inc., San Diego, CA) and combination index (CI) was calculated using CompuSyn software (ComboSyn, Inc., Paramus, NJ). The rest of the data was statistically analyzed by two-way analysis of variance (ANOVA) or Student's t test and a p value of <0.05 was considered significant.

## SUPPLEMENTARY TABLES


