# Supplementary material for: Suberoylanilide hydroxamic acid (SAHA) reverses chemoresistance in head and neck cancer cells by targeting cancer stem cells via the downregulation of nanog
Source: Genes Cancer. 2015 Mar;6(3-4):169–81. doi: 10.18632/genesandcancer.54 (PMC4426953; doi:10.18632/genesandcancer.54)
Supplement: Supplementary file 1 [file ganc-06-169-s001.pdf]

Suberoylanilide hydroxamic acid (SAHA) reverses chemoresistance in head and neck cancer cells by targeting cancer stem cells via the downregulation of nanog

Supplementary Table S1: Patient tumor characteristics

Tumor ID	Primary Site	Gender	TNM Status	Age
1	Retromolar Trigone	M	T4N0M0	72
2	Gingiva and Mandible	F	T2N0M0	58
3	Base of Tongue	F	T4N0M0	58
4	Mandible	F	T4N0M0	74
5	Mandible/Tongue	M	Unk	51
6	Mandible	M	T4N2M0	57
7	Floor of mouth	M	T4N1M0	41
8	Tonsil	M	T3N3M0	30
9	Pharynx/Larynx	F	Unk	47
10	Tonsil	M	T3N1M0	74

Supplementary Table S2: HNSCC cell line characteristics

Cell Line	Primary Site	TNM	Sex	p53	HPV
CAL 27	Tongue	T?N+	M*	Mutant	-
UM-SCC-74A	Base of Tongue	T3N0M0	M	Wild-type	-
UM-SCC-36	False Vocal Cord	T2N0M0	M	Mutant	-
UM-SCC-47	Tongue	T3N1M0	M	Wild-type	+
UD-SCC-2	Pyriform Sinus	T1N3	M	Wild-type	+
UM-SCC-10A	True Vocal Cord	T3N0M0	M	Mutant	-
UM-SCC-11B	Supraglottic Larynx	T3N0M0	M	Mutant	-
FaDu	Hypopharynx	?	M	Mutant	-

*possible loss of Y chromosome in cells?
